# Mechanisms of Quality Control in GPI-Anchored Proteins: The Central Role of the Lipid Anchor

**DOI:** 10.3390/ijms27146316

**Published:** 2026-07-16

**Authors:** Leticia Lemus, Prasanna Satpute-Krishnan, Veit Goder

**Affiliations:** 1 Department of Genetics, University of Seville, Av. Reina Mercedes, 6, 41012 Seville, Spain; llemus@us.es; 2 Sir William Dunn School of Pathology, University of Oxford, South Parks Road, Oxford OX1 3RE, UK; 3 Department of Biochemistry and Molecular Biology, Uniformed Services University of the Health Sciences, Bethesda, MD 20814, USA

**Keywords:** endoplasmic reticulum (ER), glycosylphosphatidylinositol (GPI) anchor, GPI anchor remodeling, microautophagy, RESET, post-ER quality control, vacuole/lysosome

## Abstract

Glycosylphosphatidylinositol-anchored proteins (GPI-APs) are a distinct class of eukaryotic cell-surface proteins characterized by a glycolipid anchor at their C-terminus. They display unique biophysical properties and play important roles in human diseases, including transmissible spongiform encephalopathies (TSEs), malaria, sleeping sickness, and rare disorders collectively termed inherited GPI deficiency (IGD). Because of their broad clinical relevance, GPI-APs have become a major focus of research, including their intracellular quality control (QC). Studies in diverse model organisms have revealed striking interspecies differences in GPI-AP QC pathways and notable distinctions from QC mechanisms and the degradation of misfolded species governing other secretory proteins. In this review, we summarize recent advances in the understanding of these cellular processes and propose that the observed variations in QC reflect distinct cellular strategies that balance protein QC with membrane homeostasis across evolutionary contexts.

## 1. Biogenesis of GPI-Anchored Proteins

**Figure 1:**
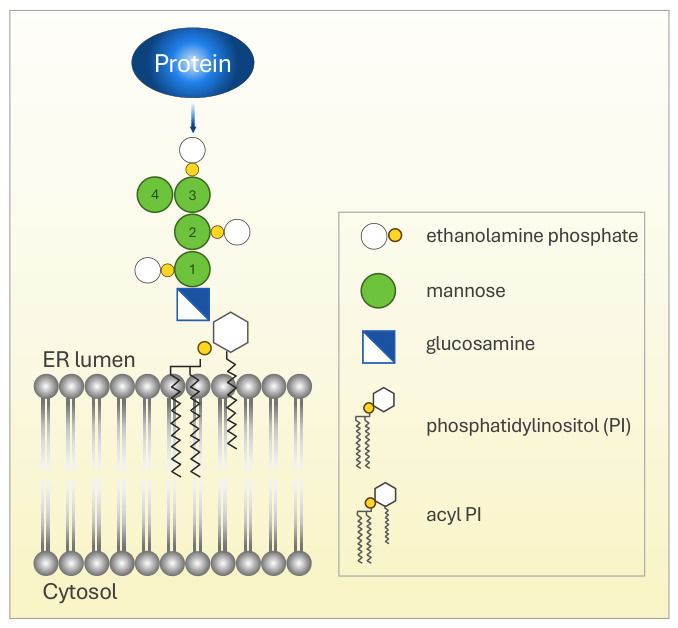
Scheme of the preassembled GPI anchor in the yeast ER. The core GPI structure Manα1-4GlcNα1-6myo-inositol-1-P-lipid is conserved across eukaryotes. Yeast GPI anchors typically contain four mannose residues, as shown (green circles 1–4), whereas most mammalian GPIs contain three. Proteins are usually linked to the GPI anchor via an ethanolamine phosphate bridge connecting the C6 hydroxyl of the third mannose to the α-carboxyl group of the protein’s C-terminal amino acid (arrow). Beyond the conserved core, GPI anchors can carry additional linear or branched glycan modifications of largely unknown function; these vary depending on the attached protein and the organism in which the anchor is synthesized. Differences also exist in the phosphatidylinositol lipid moieties, which may be modified during GPI precursor assembly (in mammals) or more generally during remodeling after protein attachment. Variations include diacylglycerol as well as lysoacyl-, alkylacyl-, and alkenylacyl-phosphatidylinositols, and in yeast many remodeled GPIs contain phosphoceramide. Furthermore, the C2 hydroxyl of inositol is initially esterified to a fatty acid, which is typically removed during the remodeling process.

Approximately 1% of all encoded proteins in protozoa, fungi, plants, and mammals undergo post-translational attachment to a glycosylphosphatidylinositol (GPI) anchor within the endoplasmic reticulum (ER) [1,2]. The biogenesis of a GPI-anchored protein (GPI-AP) is a complex, multi-step process preceded by the generation of the GPI anchor, which is preassembled by an orchestrated series of reactions at the ER membrane. The fully assembled GPI anchor consists of an inositol phospholipid and several sugar moieties (Figure 1). The biosynthesis of the GPI anchor and the subsequent protein attachment are well-characterized and have been extensively reviewed [3,4]. In brief, the generation of the GPI anchor starts with the enzymatic transfer of N-acetylglucosamine (GlcNAc) to phosphatidylinositol (PI) at the cytosolic leaflet of the lipid bilayer, generating GlcNAc-PI [5,6]. Next, GlcNAc is deacetylated, and the product GlcN-PI is flipped to the luminal leaflet of the ER membrane [7,8]. On the luminal side, inositol is acylated, generating GlcN-(acyl)PI, and in mammals, but not in fungi and plants, the diacylglycerol portion is exchanged to form a 1-alkyl, 2-acyl glycerol backbone [9,10]. Next, three mannoses are added to the GlcN in a linear fashion but with different linkage types [11,12,13]. This is followed by further modification of each mannose with a phospho-ethanolamine (PEtN) [14,15,16,17]. At this stage, a client protein in the ER lumen is coupled to the GPI anchor precursor through a transamidation reaction that is also referred to as “attachment”, coordinated by GPI transamidase, a five-subunit membrane protein complex [18,19,20]. During the attachment step, the client protein’s C-terminal transmembrane (TM) domain is removed, and the newly generated terminal carboxyl group is coupled to the amino group of the PEtN on the third mannose of the GPI anchor [21,22]. Although the mechanism of attachment appears well conserved among species, some variations in GPI anchor precursor structures have been observed. For instance, in yeast, the GPI anchor is extended with a fourth α1,2-linked mannose prior to attachment [23]. This reaction precedes the modification of the third mannose with PEtN that is important for attachment. Thus, whereas most (if not all) attached GPI anchors in yeast contain four mannoses, this GPI structure has so far only been detected in a few mammalian cell types, mostly in brain cells [23,24]. In addition, work with mutant mammalian cell lines recently showed that some proteins are preferentially attached to the PEtN of the second mannose of a GPI precursor, indicating that attachment to a GPI anchor can occur in more than one way [25].

## 2. GPI-Anchored Proteins in Physiology and Disease

Following biogenesis, GPI-APs exhibit an entirely ER-luminal topology that is preserved during ER export and Golgi trafficking, ultimately leading to their full extracellular presentation at the plasma membrane, where they function as enzymatic modifiers, adhesion molecules, immune regulators, and receptors [26,27]. Compared to transmembrane (TM) proteins, the presence of the GPI anchor makes GPI-APs form a unique class of integral membrane proteins that show specific requirements for detergent-mediated release from the lipid bilayer [28,29]. In contrast to other lipid anchors like single myristoyl, palmitoyl, and prenyl groups, the biophysical properties of the GPI anchor are thought to provide a stable membrane association for the attached protein without the need for additional protein-protein or protein-membrane interactions [30,31]. Furthermore, the complexity of the GPI anchor structure suggests cellular functions that go beyond simple membrane association [32]. It has long been known that GPI-APs tend to concentrate in specific lipid microdomains, or rafts, which affect protein trafficking and signaling [33,34]. Consequently, the biogenesis of GPI anchors and GPI-APs is tightly intertwined with membrane homeostasis and membrane traffic [28,35].

GPI-APs play critical physiological roles in both unicellular and multicellular organisms. For example, GPI-anchor biosynthesis is essential for yeast viability and for embryonic development in mice [36,37,38], and is also critical for the life cycle of trypanosomes. [39]. In yeast, many GPI-APs are released from the cell surface and become cross-linked to the cell wall, where they are covalently bound to glycans. In a transglycosylation reaction, the GPI anchor is first enzymatically cleaved between GlcN and the first mannose of the GPI anchor core structure. The liberated mannose-protein is then glycosidically linked to the nonreducing ends of cell wall glycan chains [40]. In certain protozoa, coordinated enzyme-mediated release (shedding) of GPI-APs from the cell surface is coupled to the changes in life cycle stages, such as during host switching in parasites like *Trypanosoma brucei* [41]. In mammals, the cell type-specific shedding of GPI-APs from the plasma membrane is important for development, immunity and neurogenesis, and is regulated by a variety of enzymes that are capable to cleave the GPI anchor at distinct sites [42,43,44,45,46].

GPI-APs have significant clinical relevance and are directly or indirectly implicated in several major human diseases. A prominent example is the human prion protein (PrP), which is widely expressed and whose physiological function remains incompletely defined, although initial studies suggested roles in synaptic function in the brain [47]. PrP has been demonstrated to associate with amyloid beta and mediate the internalization of amyloid beta oligomers, and this activity has been proposed to promote Alzheimer’s disease progression [48,49,50,51,52,53]. Similarly, PrP has been shown to bind to α-synuclein amyloid fibrils and hypothesized to play a role in the pathogenesis of synucleopathies, including Parkinson’s disease [54,55]. In neurons, the induced or sporadic conversion of the native, cellular form of PrP (PrP^C^) into the pathogenic, infectious form (PrP^SC^) causes the formation of amyloids and onset of Creutzfeld-Jakob and related neurodegenerative diseases. [56]. Additionally, there is a large body of work connecting PrP expression and associated signaling pathways with cancer, which has been recently reviewed [57]. Interestingly, the presence of the GPI anchor in PrP affects clinical symptoms and disease progression but the underlying mechanisms remain unclear [58,59,60].

Germline mutations in genes required for GPI synthesis in humans cause various Inherited GPI Deficiency (IGD)-related diseases, as summarized by Li et al. [61]. For example, defects in the addition of ethanolamine phosphate (EtNP) to the third mannose of the GPI anchor precursor have been associated with global developmental and intellectual delay, seizures, polymicrogyria, and peripheral neuropathy in multiple unrelated families with bi-allelic *PIG-B* mutations [62]. Comprehensive clinical reports and surveys on pathogenic variants of genes required for GPI synthesis highlight developmental delay, intellectual disability, and hyperphosphatasia—resulting from the release of cellular alkaline phosphatase—as frequent phenotypes of IGDs [63,64,65]. Paroxysmal nocturnal haemoglobinuria (PNH), a well-characterized human disease, is caused by a defect in *PIG-A*, which is essential for the initial step in GPI precursor synthesis. Dysfunctional *PIG-A* primarily affects blood cells, leading to the loss of CD55 and CD59—two GPI-APs crucial for complement regulation [66]. Additionally, impaired GPI-AP biosynthesis has been linked to several types of cancer [67].

Apart from IGDs, GPI-APs have garnered enormous attention due to their critical role in host infection by human parasites. This has fueled research aimed at understanding the underlying mechanisms of intracellular trafficking of GPI-AP to develop effective strategies against these diseases. The major surface proteins of *Plasmodium falciparum*, the causative agent of malaria, are GPI-APs that undergo frequent exchange during the parasite’s life cycle to evade immune recognition [68,69,70]. Similarly, trypanosomes—responsible for Chagas disease (*Trypanosoma cruzi*) and sleeping sickness (*Trypanosoma brucei*)—feature a dense layer of GPI-APs on their cell surface. The dependence of these parasites on their hosts, coupled with the pressures of immune system counteraction, has driven the evolution of intricate microbial life cycles and specific cellular strategies for the biogenesis and QC of GPI-AP. Consequently, trypanosomes have become pioneering model organisms for studying GPI-APs [71,72]. Recent findings indicate that certain *Trypanosoma* exhibit distinct mechanisms for the degradation of misfolded GPI-APs, differing in key steps from those observed in other model organisms and illustrating the existence of various uncharacterized cellular mechanisms directly linked to protein QC [73].

## 3. Protein Quality Control in the Secretory Pathway

Whereas protein QC in the secretory pathway was originally thought to be restricted to its point of entry, the ER, it has become clear that the eukaryotic cell harbors a variety of distinct surveillance systems in many, if not all, organelles of the secretory pathway. As will be discussed later, QC systems that operate outside the ER have specific relevance for GPI-APs.

The early detection and retention of misfolded proteins inside the ER, also known as ER quality control (ERQC), includes the unfolded protein response (UPR) and has been extensively studied and discussed [74,75,76]. In addition, it became clear that most misfolded ER proteins are eliminated by ER-associated degradation (ERAD), which involves retrotranslocation into the cytosol followed by proteasomal degradation and has been the subject of excellent recent reviews [77,78]. Certain misfolded secretory proteins that are detected inside the ER are refractory to ERAD, for instance due to the formation of mutation-specific aggregates that impede their retrotranslocation. This led to the discovery and initial characterization of several specific trafficking pathways that are used for the delivery of these proteins to the vacuole/lysosome for degradation, termed collectively ER-to-lysosome-associated degradation (ERLAD) pathways; for review see [79].

Protein misfolding can also be detected in the Golgi, in endosomes and in the vacuolar membrane, often linked to organelle homeostasis. This can be referred to as post-ERQC, for review see [80]. Work from yeast suggested early on the existence of a Golgi-localized QC [81,82]. Like in the ER, misfolded proteins can be eliminated via several cellular routes from the Golgi. Pathways named Endosome and Golgi-associated degradation (EGAD) in yeast and Golgi Apparatus-Related Degradation (GARD) in mammals promote the retrotranslocation and proteasomal degradation of misfolded and regulator proteins from the Golgi and from endosomes [83,84,85,86,87]. Alternatively, misfolded proteins or those that lack binding partners inside the Golgi are either retrieved back to the ER for ERAD or are trafficked to endosomes for subsequent delivery to the vacuole/lysosome for degradation [88,89]. Transmembrane proteins are marked for degradation in the vacuole/lysosome through ubiquitination on their cytosolic tail followed by endosomal sorting complex required for transport (ESCRT)-mediated internalization into multivesicular bodies (MVBs), for review see [90]. Misfolded soluble proteins destined for vacuolar/lysosomal degradation are sorted from the Golgi to endosomes by the conserved cycling receptor Vps10/sortilin. It has been shown to bind unfolded proteins through its luminal 1 domain [91,92,93]. Work from yeast has demonstrated that this pathway has relevance for the QC and degradation of misfolded GPI-APs [94].

## 4. GPI Anchor Remodeling and Implications for GPI-AP QC

The lack of a transmembrane domain makes GPI-APs unique membrane-attached cell surface proteins with no exposure to the cytosol. All intracellular trafficking of GPI-APs therefore depends on transmembrane (TM) adaptor proteins that connect the luminal or extracellular protein with the cytosolic machinery that regulates membrane traffic. For instance, the internalization of GPI-APs from the plasma membrane into endosomes for their regulated turnover by vacuolar/lysosomal degradation depends on cycling crossing-over suppressor (cos) proteins, which constitute a conserved class of TM adaptor proteins [95]. Similarly, the initial export from the ER depends on adaptor proteins classified as p24 proteins, which form heteromeric complexes [96,97,98,99]. p24 proteins bind to GPI-APs on the luminal side of the ER membrane, while their cytosolic tails interact with the COPII machinery to facilitate the concentration of GPI-APs at ER exit sites (ERES) [100].

The binding of GPI-APs to p24 proteins is regulated by GPI anchor remodeling, a process that includes modifications on both sugar and lipid moieties of the GPI anchor. Remodeling starts usually after the attachment of proteins to the preassembled GPI anchor precursor, although exceptions exist [101]. Soon after attachment, the acyl chain linked to inositol is removed by a conserved ER-resident deacylase—Bst1 in yeast, PGAP1 in mammals, or PtPGAP1 in plants [102,103]—with a few exceptions reported, such as in human erythrocytes [104]. In yeast, either the acyl chain at the sn-2 position of the diacyl glycerol is then exchanged for a long saturated C-26 fatty acid by the ER-resident enzymes Per1 and Gup1 or, alternatively, the diacylglycerol moiety is exchanged entirely for a ceramide by the ER-resident remodelase Cwh43 [105,106,107]. In mammalian cells, lipid remodeling on the 1-alkyl, 2-acyl glycerol backbone occurs only after ER export, in the Golgi, for review see [108]. Prior to ER export, the PEtN on the second mannose of the GPI anchor is removed by a conserved ER-resident deacetylase, Ted1 (yeast) or PGAP5 (mammals) [109,110]. In addition to enhancing the binding of GPI-APs to p24 proteins, GPI remodeling also promotes their prior concentration within sphingolipid-enriched microdomains. Together, these processes coordinate the efficient export of GPI-APs from the ER [109,111,112,113,114].

Protein folding and GPI anchor remodeling occur concurrently within the ER, raising a key question: to what extent are these complex processes functionally interconnected and co-regulated? For example, does terminal GPI anchor remodeling take place only after proteins have successfully folded and passed ERQC? Early insights from studies in yeast and mammalian cells revealed that misfolded GPI-APs are predominantly—though not exclusively—degraded via vacuolar or lysosomal pathways, rather than through ERAD [115,116,117,118]. Interestingly, in yeast mutants that block ER protein export, ERAD became the predominant pathway for the degradation of misfolded GPI-APs [118]. These findings demonstrated that misfolded GPI-APs are generally ERAD-competent—contrary to the notion that the GPI anchor might present a steric barrier to retrotranslocation [119]. The observation that, under normal conditions, these proteins are frequently exported from the ER and subsequently degraded in vacuoles or lysosomes supports a model in which GPI anchor remodeling—rather than protein folding—determines the kinetics of ER export. Accordingly, at least in yeast, protein folding and GPI anchor remodeling appear to operate independently, with misfolded GPI-APs relying predominantly on post-ERQC mechanisms for their disposal [94,118,120].

These mechanisms, however, do not appear to be universal. Comparative analyses of yeast data alongside both established and emerging findings from other model organisms reveal shared features as well as striking differences in the strategies used by the eukaryotic cell to handle misfolded GPI-APs. In the sections that follow, we summarize and discuss the current understanding of GPI-AP QC across yeast, mammals, plants, and protozoa.

## 5. Mechanisms of GPI-AP QC in Different Model Systems

### 5.1. Yeast

A prominent model misfolded GPI-AP for studying its QC in the yeast *S. cerevisiae* is Gas1*, the G291R mutant of the β-1,3-glucanosyltransferase Gas1 involved in cell wall synthesis [121]. Gas1* is degraded with a half-life of 45–60 min [118,121]. In wild-type cells, Gas1* is degraded mainly inside the vacuole, whereas a minor fraction is routed to Hrd1-dependent ERAD [118]. Like Gas1*, other chimeric or non-related misfolded GPI-APs, such as CPY*GPI or Cwp2*, are routed predominantly to the vacuole for degradation [94,118]. Interestingly, the fraction of Gas1* that is targeted to ERAD depends in part on protein *O*-mannosylation, a modification of serine and threonine side chains with single mannose moieties inside the ER that was previously known to have functional importance for ERQC and ERAD [120,121,122,123,124]. Gas1*, but not Gas1, becomes *O*-mannosylated by ER-resident protein mannosyltransferases Pmt1 and Pmt2, which form a heterodimeric complex and have a chaperone function in addition to their mannosyltransferase activity [120]. The Pmt1/2 complex interacts physically with components of the ER export machinery for GPI-APs as well as with the Hrd1 ERAD machinery, and its deletion shifts Gas1* degradation entirely to vacuolar degradation [115,120]. These findings suggest that the Pmt1/2 complex retains Gas1* inside the ER to facilitate folding and can shuttle a subpopulation of terminally misfolded Gas1* to ERAD. The fact that most cellular Gas1* is degraded inside the vacuole rather than by ERAD does not result from the strong endogenous expression level of Gas1* and/or saturation of the ERAD pathway because efficient “rerouting” to ERAD can be observed when its ER export is blocked genetically [118]. In addition, Gas1* is degraded almost entirely by ERAD in wild-type cells if its attachment to a GPI anchor is prevented by a single point mutation that abolishes recognition by the transamidase complex [118]. Collectively, these results show that the presence of the GPI anchor is the cause for ER export and predominant vacuolar degradation of Gas1*. It also argues that the GPI anchor itself is not a structural obstacle for protein retrotranslocation and ERAD. How ERAD of a GPI-AP occurs mechanistically and what happens to the GPI in this process is still unclear, but some relevant information might come from studies in protozoa (see below).

**Figure 2:**
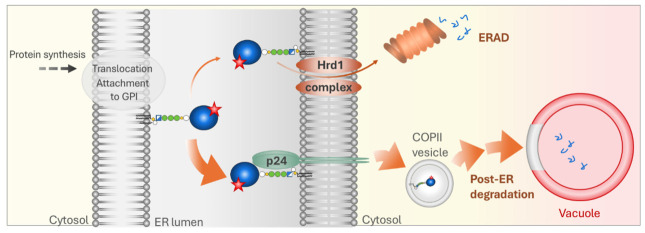
The two degradation pathways for misfolded GPI-APs in yeast. After protein synthesis and translocation into the ER, the original C-terminal transmembrane domain is removed, and the protein is attached to a preassembled GPI anchor. Two degradation pathways are known in yeast and are used to different extents, as illustrated by the different arrow thicknesses. Top half: a variable but generally minor fraction of misfolded GPI-APs (red star indicates misfolding) is routed to ERAD. It remains unknown what happens to the GPI anchor during retrotranslocation of GPI-APs via the Hrd1-complex. Bottom half: Following attachment, the GPI anchor undergoes remodeling—a process that is independent of protein folding and occurs even on misfolded GPI-APs. The remodeled GPI anchor acts as a strong ER export signal and promotes p24-dependent vesicular export of misfolded GPI-APs. Once exported, these proteins are subject to post-ERQC and are ultimately directed to the vacuole for degradation (peptides resulting from protein degradation, depicted in blue within the vacuole). Major components and mechanisms governing this post-ER degradation pathway have recently been elucidated.

Why then is Gas1* exported in large quantities from the ER rather than being retained and routed to ERAD? It was found that Gas1 and Gas1* bind with the same efficiency to the p24 family member Emp24, the adaptor for connecting GPI-APs with the cytosolic COPII machinery for ER export [118]. At the same time, binding was equally abrogated in mutant cells that prevent GPI anchor remodeling [118]. These data showed that the GPI anchors of correctly folded Gas1 and misfolded Gas1* are remodeled with indistinguishable efficiencies in vivo and support the notion that GPI anchor remodeling is not functionally connected to protein folding in yeast. From the model systems that have been studied so far, yeast appears to provide the most limited ERQC for GPI-APs (Figure 2). Possible reasons for this will be discussed in the last section.

**Figure 3:**
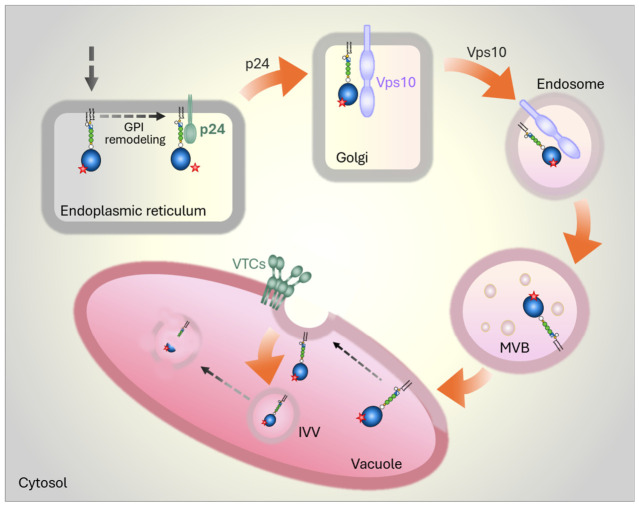
A detailed depiction of the post-ER degradation pathway for misfolded GPI-APs in yeast. Misfolded GPI-APs (indicated with a red star) are rapidly exported from the ER via the p24-complex and transported to Golgi. There, they are diverted from the early secretory pathway and routed to endosomes in a post-ERQC step that requires Vps10. At endosomes, misfolded GPI-APs are not internalized into intraluminal vesicles but instead remain on the limiting membrane of multivesicular bodies (MVBs). Upon fusion of MVBs with the vacuole, these proteins are delivered to the vacuolar membrane and become exposed to the vacuolar lumen—a transient intermediate that accumulates in Δ*pep4* cells. From the vacuolar membrane, misfolded GPI-APs are internalized through a process resembling microautophagy and dependent on the vacuolar transporter chaperone (VTC) complex. The resulting intravacuolar vesicles (IVVs) containing misfolded GPI-APs are ultimately degraded in an Atg15-dependent manner. The regulation of vacuolar membrane invagination during this internalization process, and the specific mechanistic role of the VTC complex, remain open questions.

GPI-APs in yeast are therefore bona fide substrates for post-ERQC. Recently, the trafficking pathway of Gas1* from the ER to the vacuole has been identified [94] (Figure 3). ER-exported Gas1* is routed to the Golgi apparatus, from where it is diverted to endosomes in a step that requires the conserved transmembrane protein Vps10/sortilin, whereas wild-type Gas1 is trafficked from the ER to the Golgi and further to the plasma membrane. Thus, very similar to the handling of soluble misfolded proteins in the Golgi, Vps10/sortilin-dependent sorting appears to be a critical post-ERQC step for misfolded GPI-APs. What elements of Gas1* are recognized at this stage remains a question of ongoing investigation. Another important finding concerns the uptake of Gas1* by the vacuole [94]. Gas1* fails to internalize from endosomes, probably because there are no adaptors present that could be ubiquitinated by the cytosolic ubiquitination machinery and could provide the crucial link for recruiting the ESCRT machinery for membrane invagination and formation of Gas1*-containing multi-vesicular bodies [90]. Instead, after fusion of endosomes with the vacuole, Gas1* is transiently present at the vacuolar membrane, facing the lumen of the vacuole. From there it is internalized into intravacuolar vesicles in a yet poorly defined process that is regulated by members of the vacuolar transport chaperone (VTC) family [94]. VTCs are multispanning vacuolar membrane proteins with previously described functions in microautophagy [125]. Interestingly, internalization of Gas1* is inhibited in a Δ*pep4* mutant, a phenotype that mimics that observed for the vacuolar uptake of lipid droplets and correlates with the inhibition of the formation of specialized lipid microdomains in the vacuolar membrane [126,127]. This could indicate that cargo sequestering into vacuolar membrane microdomains with raft-like properties and with affinity to GPI anchors is part of the mechanism that mediates Gas1* internalization from the vacuolar membrane, but precisely how it works remains an important open question [128]. Although the protein part of Gas1* could be degraded directly from the vacuolar membrane by vacuolar proteases, internalization ensures that the GPI-AP is degraded in its entirety, including its remodeled GPI anchor, which contains long chain fatty acids or ceramide. Thus, internalization of most (if not all) Gas1* into intravacuolar vesicles prior to degradation might also be required to maintain vacuolar membrane homeostasis.

### 5.2. Mammalian Cells

A large body of work addressing the QC and degradation of GPI-APs in mammalian cells comes from studies with human PrP. Proteasomal degradation of wild-type and disease-associated misfolded variants of human PrP was observed when GPI attachment was prevented either by introducing mutations into genes encoding GPI-anchor biosynthetic machinery or by mutating the GPI-anchor attachment site in PrP [116]. In contrast, when GPI-anchoring was occurring normally, the same species were ultimately routed to lysosomes for degradation [116,129,130,131,132,133]. These findings are analogous to those obtained with yeast where misfolded GPI-AP are predominantly trafficked to the vacuole for degradation [118]. Initial conflicting data suggesting that presumed GPI-AP are susceptible to proteasomal degradation in mammalian cells came from protein populations that failed to be targeted to the ER and therefore lacked a GPI-anchor [129,134,135]. The observed inefficient ER targeting of PrP was found to be due to its weak N-terminal signal peptide in comparison to that of other proteins, such as for example prolactin or osteopontin [130,131,132].

The trafficking pathway by which misfolded GPI-APs PrP move from the ER to lysosomes was dissected using PrP*, a C179A variant of PrP which causes misfolding due to disruption of disulfide bond formation and furthermore contains the N-terminal prolactin signal peptide for efficient translocation [117]. Additionally, a version of PrP* containing a yellow fluorescent protein (YFP)-tag encoded immediately upstream of the PrP mature domain and downstream of the prolactin signal peptide was constructed to allow for live-cell imaging. Expression of YFP-PrP* in normal rat kidney (NRK) cell culture produced a major ER-localized population and a minor population that could be detected in lysosomes at steady-state [117]. At the same time, YFP-PrP* constitutively underwent ER-export and was trafficked to lysosomes via the plasma membrane. Transient exposure to the plasma membrane was revealed through anti-YFP antibody-uptake assays or through forced trapping at the plasma membrane with endocytosis inhibitors [117,136,137]. Interestingly, the chemical ER stressors thapsigargin or dithiothreitol (DTT), each induced a rapid release of YFP-PrP* from the ER and near-synchronous ER export [117]. In NRK cells, treatment with thapsigargin resulted in the traffic of most YFP-PrP* to the Golgi within 30 min and to the lysosomes within 60 min, and near complete degradation within 90 min [117,138]. Measurements of total fluorescence intensity of YFP-PrP* over 30 min after addition of thapsigargin indicated that at least 85% of the YFP-PrP* molecules moved from the ER to the Golgi [117]. A quantitative approach involving flow cytometry analysis of green fluorescence protein (GFP)-PrP* molecules expressed in Flp-in T-Rex human embryonic kidney (HEK) 293 cells demonstrated that at least 85% of GFP-PrP* gained exposure on the plasma membrane on their way to lysosomes [136]. The observed ramped up ER export of PrP within minutes of treatment with thapsigargin or DTT, well before the transcriptional activation of the UPR, suggested that this mechanism may constitute a fast cellular response for maintaining ER homeostasis during acute stress through clearance of a subset of misfolded proteins [117]. The pathway was therefore named “Rapid ER Stress-induced Export” or the “RESET” pathway [117]. Supporting a general role of this pathway, other GPI-APs, such as additional human disease-associated variants of PrP, CD59, folate receptor, Thy1, decay accelerating factor, and an artificial misfolding protein construct comprising the luminal domain the transmembrane ERAD substrate TCR alpha appended to a GPI-anchor, were shown to undergo thapsigargin-induced RESET with similar kinetics to YFP-PrP* in NRK cells [117,139]. RESET of YFP-PrP* was also observed in other cell types, including mouse neuroblastoma neuro-2 (N2a), HeLa, COS-7, IB3-1 and HEK293T cells, suggesting widespread conservation and was found to be facilitated by calnexin and members of the p24-family [117,136,137,138,139]. How misfolded GPI-APs are recognized at the plasma membrane for endocytosis in presence or absence of binding partners is still unknown. Interestingly, the population of exiting PrP* in Flp-in T-Rex HEK293 cells showed an invariant interactome including members of the p24-family, and the ER chaperones BiP and calnexin during traffic from the ER to the plasma membrane [136]. It was proposed that exposure of the misfolded GPI-AP together with the chaperones at the plasma membrane provides a signal for subsequent endocytosis prior to lysosomal degradation [136].

**Figure 4:**
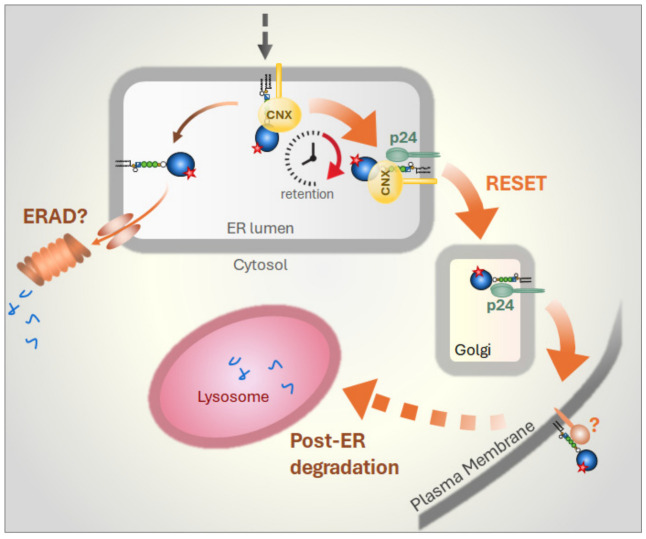
Degradation pathways for misfolded GPI-APs in mammalian cells. Several studies have demonstrated a specific role for calnexin (CNX)-dependent ERQC in retaining misfolded GPI-APs (indicated with a red star) in the ER. GPI anchor remodeling has also been linked to associations with calnexin and calreticulin, providing a physical connection between anchor remodeling and protein folding. Under conditions of ER stress, misfolded GPI-APs are released from ER-resident chaperones, leading to their rapid p24-dependent export from the ER to the Golgi, by a pathway that has been termed “Rapid ER Stress-induced Export” (RESET). In contrast to yeast, misfolded GPI-APs in mammalian cells are trafficked from the Golgi to the plasma membrane before being internalized and ultimately delivered to lysosomes for post-ER degradation (peptides resulting from protein degradation, depicted in blue within the vacuole). Separate assays assessing the surface exposure of PrP* suggested that it might be present at the plasma membrane in complexes with normally ER-resident proteins, including calnexin and p24-family members, which has been proposed to facilitate endocytosis. The precise trafficking itinerary from the cell surface to lysosomes remains unclear. Similar to yeast, little to no degradation of misfolded GPI-APs has been demonstrated to occur via ERAD.

Data coming from work with the GPI-APs CD55 and CD59, both of which form part of the complement regulatory system and cause paroxysmal nocturnal hemoglobinuria if defect, revealed additional insight into the QC of this class of proteins [140,141,142]. Misfolding CD59*, which is characterized by a C94S mutation, was retained in the ER. At the same time, simultaneous deletion of both calnexin and calreticulin led to a faster ER exit and resulted in inefficient deacetylation of inositol on its GPI anchor by PGAP1 [143,144]. In addition, calnexin was found to physically interact with PGAP1 [143]. These data prompted the hypothesis that N-glycan-dependent ER retention of misfolded GPI-APs through calnexin and calreticulin can prevent premature GPI anchor remodeling and ER export [139,143,144,145]. This differs from the scenario described in yeast and suggests that mammalian cells do possess a certain degree of physical and functional connection between protein folding and GPI anchor remodeling (Figure 4). This would allow for an increased time window for the ERQC of GPI-APs.

### 5.3. Plants

Almost all genes involved in the biogenesis of the GPI anchor and protein attachment to it in mammalian cells have homologs in the plant model *Arabidopsis thaliana* and initial studies have shown that they play comparable roles [146,147]. Likewise, the role of GPI lipid remodeling and p24 family proteins in the ER export of GPI-APs is conserved in plants [100,103]. Recent work with the model organisms *Nicotiana benthamiana* and *Arabidopsis thaliana* has started to provide insight into ERQC of GPI-APs in the plant kingdom [148]. The expression of misfolding mutants of the GPI-anchored lipid transfer protein G1 (LTPG1) led to their routing to the vacuole for degradation. Additional mutations that prevented the attachment to the GPI anchor resulted in targeting the same protein to ERAD. Overall, the results are in good agreement with those obtained with yeast and mammalian cells: a terminally misfolded GPI-AP is predominantly routed to the vacuole/lysosome for degradation.

**Figure 5:**
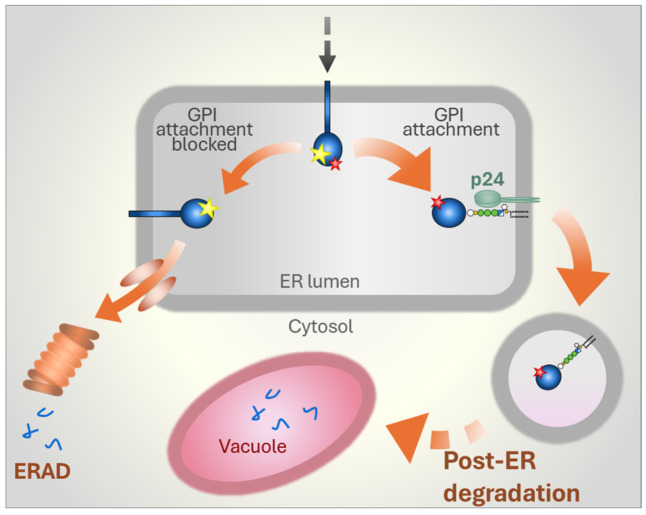
Degradation pathways for misfolded GPI-APs in plants. Current evidence suggests that, as in yeast, most misfolded GPI-APs (red star) in plant cells are routed to the vacuole for degradation (peptides resulting from protein degradation, depicted in blue within the vacuole) through post-ERQC pathways that remain largely unexplored. The available data further indicate that proteins containing severely misfolded domains (yellow star) are prevented from being attached to preassembled GPI anchors within the ER. This preemptive form of ERQC preserves the proteins’ original C-terminal transmembrane domain and enables efficient ERAD of these species. The degree to which this pathway is used is not known. In addition, whether such a preemptive ERQC mechanism for GPI-APs operates in yeast or mammalian cells remains unknown.

Interesting insights into additional QC mechanisms for GPI-APs came from studying chimeric proteins where a C-terminal domain that contained a site for GPI anchor attachment was fused with different types of misfolding N-terminal domains. Whereas most mutants were routed to the vacuole for degradation, a mutant containing the misfolding domain of STRUBBELIG (SUBEX-C57Y) was routed to ERAD instead [148]. Detailed analysis revealed that this was due to failure of the chimeric protein to become attached to a GPI anchor, explaining their routing to ERAD. Of note, removing the misfolded domain from the fusion proteins restored GPI anchoring. These results were used to suggest that ERQC precedes protein attachment to a GPI anchor and is capable of preventing attachment in case of certain types of misfolding [148,149] (Figure 5). It was further suggested that the degree or “severity” of misfolding was critical for allowing or preventing attachment. Although failure of attachment could be caused by a variety of factors unrelated to protein QC mechanisms, these data provide useful hypotheses for further testing. It appears plausible that rapid protein aggregation inside the ER or failure to attract chaperones that promote folding could inhibit attachment to a GPI anchor. Early detection and degradation of a terminally misfolded protein via ERAD would be more energy-efficient than degrading the same protein after attachment of a metabolically expensive GPI anchor and subsequent trafficking to the vacuole. Why only certain types of misfolded proteins abort attachment to a GPI anchor, and what distinguishes these misfolding variants, remain important open questions.

### 5.4. Protozoa

One of the most relevant human parasites, the malaria-causing *Plasmodium falciparum*, has most of its cell surface covered with GPI-APs, which are intensely studied for the roles in virulence and immune defense [68]. Most insights into the QC of GPI-APs in protozoa came from studies related to *Trypanosoma brucei*, which causes sleeping sickness in humans. Research on this parasite has been paramount for the discovery and elucidation of the GPI structure [71]. The major protein on the cell surface of the parasite in the bloodstream form is the GPI-AP VSG (variant surface glycoprotein). During each new life cycle, VSG is synthesized as a variant due to expression switching between up to 2000 different VSG-encoding genes that are clustered at distinct expression sites as well as due to gene conversion during recombination events. Both mechanisms contribute to virulence because VSG variants protect the parasite from the immune system of the host [41]. Due to the constant and massive synthesis of VSG, failure to rapidly detect and eliminate misfolded or aggregation-prone VSG variants by efficient ERQC or post-ERQC systems was predicted to be lethal to the parasite. This sparked an interest in studying mechanisms of QC of GPI-APs in *T. brucei*. Initial studies showed that the ER exit of VSG and trans-sialidase, another GPI-AP in *T. brucei*, was significantly slowed down if GPI anchoring failed [150]. Reversely, the ER exit was accelerated if an otherwise slowly secreted soluble protein was attached to a GPI anchor [151]. These data agree with the scenarios found in other eukaryotes and confirm that GPI anchors are bona fide ER export signals.

Recently, the QC and degradation mechanisms of the VSG-related high-affinity transferrin receptor (TfR) in *T. brucei* have been investigated in detail [152]. The TfR is formed by heterodimerization of two subunits within the ER: ESAG6, a GPI-anchored protein, and ESAG7, a transmembrane protein. When a misfolded version of ESAG6 (HA:E6) was expressed, it accumulated as a cytosolic intermediate upon proteasome inhibition. Interestingly, this intermediate was recognized by an antibody specific for GPI anchors lacking lipid moieties [152]. This suggests that the HA:E6 was fully translocated into the ER and has been attached to the GPI anchor, before it was recognized by ERQC and retrotranslocated back into the cytosol as part of ERAD. Whereas experiments with yeast suggested that misfolded GPI-APs can be targeted to ERAD, it was unclear if and how the GPI anchor is removed during this process. The experiments with HA:A6 suggest that the removal of GPI lipids might be linked to ERAD of GPI-APs. Where and how the lipids of the GPI anchor are removed is currently unclear and poses relevant mechanistical and topological questions. Interestingly, *T. brucei* strongly expresses a cytosolic GPI-specific phospholipase C (GPI-PLC). It has long been thought to have its major function in releasing VSG from the cell surface (shedding) of the bloodstream form of the parasite upon host switching, although the topological paradox (cytosolic GPI-PLC versus extracellular VSG) has been difficult to reconcile with the proposed mechanism. Results from work addressing this issue suggested that the process involves the translocation of GPI-PLC into the extracellular space mediated by reversible acylation or by secretion in extracellular vesicles but the contribution of either pathway to VSG shedding is not clear [153,154]. In addition, a recent study suggests that GPI-PLC has only a minor role in VSG shedding and is more important for mediating VSG endocytosis [155]. The data linked to QC of GPI-APs now suggest that GPI-PLC might have an important cellular function in removing GPI lipids of retrotranslocated GPI-APs prior to proteasomal degradation [152]. The authors propose that retrotranslocated HA:A6 has to be completely extracted from the ER membrane together with its GPI anchor for the reaction to occur [152]. In an alternative scenario, GPI-PLC-mediated cleavage of the GPI anchor could occur on a retrotranslocated HA:A6 with its GPI anchor flipped to the cytosolic side of the ER membrane. This would avoid exposure or shielding of lipids to the cytosol. To resolve these questions, further experiments are needed.

**Figure 6:**
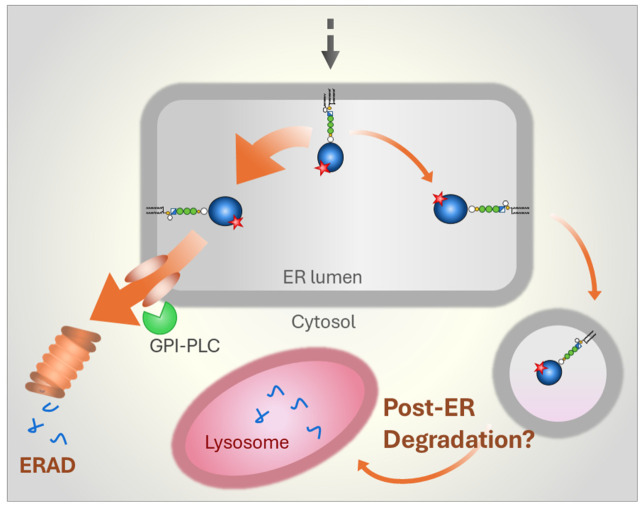
Degradation pathways for misfolded GPI-APs (red star) in protozoa. Data from the parasite *T. brucei* indicate that misfolded GPI-APs are efficiently routed to ERAD, with little to no protein directed to lysosomes (peptides resulting from protein degradation, depicted in blue within the vacuole) for post-ER degradation. The requirement to synthesize large quantities of GPI-APs at specific life cycle stages, combined with the need for stringent QC, may have driven the evolution of a dedicated ERAD branch for disposing of misfolded GPI-APs. A key component of this pathway appears to be a highly abundant, GPI-specific phospholipase C (GPI-PLC) present in the cytosol of *T. brucei*. The presence of similarly abundant GPI-APs on the surface of other parasites suggests that this efficient ERAD mechanism may be common among certain protozoa. Whether ERAD of misfolded GPI-APs in other organisms—such as yeast or mammalian cells—also relies on specific lipases or instead employs distinct mechanisms remains an open question. (Thicker lines indicate greater pathway usage).

In summary, host-switching parasites, such as *T. brucei,* might have evolved a highly specialized cellular machinery for the rapid and efficient detection and elimination of misfolded GPI-APs (Figure 6). In *T. brucei*, such a need could be based on the fact that the parasite produces up to an estimated 30.000 VSG molecules/min [156]. A highly abundant GPI-specific PLC might be part of an ERAD machinery with a specific branch for GPI-APs.

## 6. Integrative Model and Outlook

The biophysical properties of the GPI anchor have long been known to facilitate the concentration of GPI-APs in specific membrane domains, or rafts. This feature, together with an entirely luminal topology of the protein part, makes GPI-APs special in that they depend on lipid-based sorting and adaptor-dependent trafficking inside the cell. The studies presented in this review showcase that the QC of newly synthesized GPI-APs is equally special. Unlike most other proteins of the secretory pathway, misfolded GPI-APs are usually no ERAD substrates and rely on post-ERQC and on vacuoles/lysosomes for degradation. In addition, there are organism-specific differences and notable exceptions. We propose that all these findings reflect a tight functional connection between the QC of GPI-APs and ER membrane homeostasis, with the observed variations in part linked to differences in GPI-anchor remodeling.

In yeast, GPI anchor lipid remodeling is occurring exclusively inside the ER, including the addition of long chain fatty acids or ceramide. Accumulation of remodeled GPI-APs upon retention of misfolded species inside the ER would result in ER membrane stress [157,158]. This has been indirectly demonstrated by the deletion of p24 family members dedicated almost exclusively to the ER export of GPI-APs, which resulted in robust induction of the UPR [159]. Thus, ER-localized GPI lipid remodeling in yeast leads to a rapid export of GPI-APs from the ER, even if misfolded, and makes GPI-APs substrates for post-ERQC.

In analogy, the fact that in mammalian cells lipid remodeling of GPI anchors occurs mostly in the Golgi rather than in the ER can explain why GPI-APs can be retained inside the ER for a larger time window compared to yeast, at least under non-stressed conditions. The retention of incorrectly folded, non-(lipid)-remodeled GPI-APs in the ER likely imposes a tolerable or basal level of ER membrane stress. Only in case ER stress is further enhanced, including by drugs such as dithiothreitol or thapsigargin, rapid ER export of GPI-APs through the RESET pathway is observed, leading to rapid lysosomal degradation of misfolded species under these conditions [117,158,160]. Additional support for a tight connection between GPI-APs and ER membrane homeostasis in mammalian cells comes from the finding that accumulation of unanchored GPI anchors in the ER membrane triggers suppression of de novo GPI-anchor synthesis through degradation of the biosynthetic machinery [161]. Nevertheless, prolonged retention of even small amounts of misfolded GPI-APs in the ER—levels that may be insufficient to trigger RESET—could become deleterious over time. For example, neuronal death in prion diseases has been partly attributed to chronic ER stress induced by misfolded PrP mutants [162].

Data from plants further illustrates the diversity of evolutionary solutions for regulating GPI-AP QC in connection with ER membrane homeostasis. Rapid and rigorous ERQC was shown to prevent attachment of terminally misfolded proteins to GPI anchors in the first place, thereby eliminating potential ER membrane stress caused the ER retention of misfolded GPI-APs.

It is interesting to observe that the host switching parasite *T. brucei* employs an entirely different modus operandi for the ERQC of GPI-APs. It might be linked to the unique biology of this and related parasites, which involves the frequent generation of new variants of VSG, the most abundant GPI-AP that makes up almost all protein on the cell surface and is paramount for the evasion of the host immune system and thus for cellular survival.

The extraordinarily high level of expression might have promoted the co-evolution of an ERAD pathway for the efficient elimination of excess molecules [163]. However, simple protein abundance was ruled out as a sole driver of ERAD, since only misfolding mutant versions, but not wild-type VSG, were ERAD substrates [164]. Another important factor contributing to efficient ERAD could be the structure of the N-glycans synthesized in *T. brucei*. In contrast to higher eukaryotes, the parasite belongs to a group of protists that generates N-glycans lacking terminal glucose residues and containing fewer mannose residues, including N-glycans with a Man_5_GlcNAc_2_ structure, which in higher eukaryotes is associated with ERAD targeting following mannose trimming [165,166]. Although *T. brucei* contains a Yos9 homolog, the lectin that recognizes Man_5_GlcNAc_2_ during ERAD in higher eukaryotes, the process must be regulated differently in the parasite, since wild-type VSG is decorated with Man_5_GlcNAc_2_ without being constitutively routed to ERAD [167]. These findings suggest that the mechanisms governing substrate recognition and commitment to ERAD have diverged substantially between trypanosomatids and higher eukaryotes.

Yet another hypothesis for why the degradation of GPI-APs in this organism is linked to ERAD can be the composition of the GPI anchor. The lipid moieties of the VSG GPI anchor consist exclusively of myristic acid and are therefore unusually short compared to anchors in other cells [168]. This might negatively affect the association of VSG with membrane rafts and the ability to trigger ER membrane stress upon ER retention. Although many questions concerning the mechanism of ERAD of misfolded VSG, including the membrane topology of intermediates, remain open, the contribution of the specific lipase GPI-PLC in this process serves as a valuable working hypothesis and should be rigorously investigated further.

The observed variations in ERQC and degradation of misfolded GPI-APs might not be exclusive. The use of only a few model proteins in each system might generate a misleading bias. It is possible that all modes of ERQC and degradation of GPI-APs are found in all eukaryotic cells and utilized to a certain degree, with each cell type having preferred mechanisms and pathways in place in adaptation to its biology. In support of such a scenario, yeast can route misfolded GPI-APs to ERAD when ER exit is genetically blocked. Conversely, ER retention of misfolded GPI-APs in mammalian cells is rapidly relieved upon ER stress, allowing their export from the ER. Together, these observations indicate that both systems retain flexibility and can respond rapidly to changing environmental conditions. In fact, the results from work with parasites, where ERAD has been found to preferentially mediate the degradation of misfolded GPI-APs, provide an excellent starting point for investigating ERAD mechanisms for GPI-APs in other model systems. In particular, they may help address important mechanistic questions, such as the fate of the GPI anchor before or after retrotranslocation and membrane extraction before degradation.

## Data Availability

No new data were created or analyzed in this study. Data sharing is not applicable to this article.
